# Profils clinicobiologiques et pronostiques du myélome multiple: à propos de 60 cas

**DOI:** 10.11604/pamj.2025.50.40.46075

**Published:** 2025-02-03

**Authors:** Wiem Lazzem, Meriem Belhédi, Souhir Krichen, Sonia Chouaieb

**Affiliations:** 1 Service des Laboratoires, Hôpital Habib Thameur Tunis, Tunis, Tunisie

**Keywords:** Myélome multiple, gammapathie monoclonale, diagnostic, pronostic, Multiple myeloma, monoclonal gammopathy, diagnosis, prognosis

## Abstract

Le myélome multiple (MM) est une dyscrasie plasmocytaire médullaire maligne liée à la sécrétion d´une immunoglobuline (Ig) monoclonale. L´objectif de cette étude est de décrire les particularités épidémiologiques cliniques, biologiques et pronostiques d´une série de patients ayant un myélome multiple, colligés au Service des Laboratoires de l´Hôpital Habib Thameur de Tunis. Il s´agit d´une étude descriptive rétrospective réalisée au Service des Laboratoires de l´Hôpital Thameur de Tunis sur une période de 10 ans (2003-2023), incluant les patients atteints de MM. Le recueil et l´analyse des données ont été effectués par la consultation des dossiers et l´utilisation du logiciel Excel 2010. Soixante patients ont été inclus. L´âge moyen était de 67,61±8,7 ans, le sex-ratio masculin/féminin était de 0,76. Les douleurs osseuses étaient la circonstance de découverte la plus fréquente, dans 75% des cas. Les anomalies radiologiques étaient retrouvées dans 50 cas (83%) dominées par l´atteinte du rachis (57%). Sur le plan biologique ont été notées: une anémie chez 74% des patients, une thrombopénie chez 17% des patients, et une vitesse de sédimentation (VS) accélérée dans 90% des cas. Le myélogramme a permis le diagnostic dans 70% des cas. L´immunoglobuline monoclonale était une IgG (62%), une IgA (22%), une IgM (2%), et une chaîne légère (12%). Selon la classification de Durie et Salmon, la majorité de nos patients (74%) étaient au stade III. Cette étude a permis de mieux cerner les caractéristiques épidémiologiques, cliniques et biologiques du MM. Malgré les progrès significatifs réalisés depuis plus de 20 ans, le myélome reste une pathologie de mauvais pronostic.

## Abstract (en)

Multiple myeloma (MM) is a malignant bone marrow plasma cell dyscrasia, associated with the secretion of a monoclonal immunoglobulin (Ig). The purpose of this study is to describe the epidemiological, clinical, biological and prognostic features of a cohort of patients with multiple myeloma, whose data were collected at the Laboratory Department of Habib Thameur Hospital in Tunis. We conducted a retrospective descriptive study of patients with MM in the Laboratory Department of Habib Thameur Hospital in Tunis over a period of 10 years (2003-2023). Data collection and analysis were performed using patient records and Microsoft Excel 2010 software. A total of 60 patients were included, with a mean age of 67.61 ± 8.7 years and a male-to-female ratio of 0.76. Bone pain was the most common presenting symptom, occurring in 75% of cases. Radiological abnormalities were identified in 50 patients (83%), predominantly affecting the spine (57%). Laboratory tests showed anemia in 74% of patients, thrombocytopenia in 17% of patients, an elevated erythrocyte sedimentation rate (ESR) in 90% of cases. Myelogram confirmed the diagnosis in 70% of cases. The monoclonal immunoglobulin identified was IgG in 62% of cases, IgA in 22%, IgM in 2%, and light chain in 12%, one patient presented with biclonal MM. According to the Durie and Salmon classification, the majority of patients (74%) were diagnosed at stage III. This study provides a better understanding of the epidemiological, clinical, and biological characteristics of MM. Despite significant advances made over the past two decades, multiple myeloma remains a disease with a poor prognosis.

## Introduction

Le myélome multiple (MM) est une dyscrasie plasmocytaire médullaire maligne liée à la sécrétion d´une immunoglobuline (Ig) monoclonale. C´est la plus fréquente des gammapathies malignes [1]. Les manifestations polymorphes, sont la base de critères diagnostiques qui ont été réactualisés en 2014 [2]. Cette révision des critères diagnostiques autorise le traitement de certains patients asymptomatiques, sur des critères biologiques et radiologiques [3]. Les connaissances sur l´épidémiologie descriptive du MM en Tunisie sont rares. Plusieurs études ont été faites à l´échelon de certaines villes, mais elles restent éparpillées. La survie médiane n´excède pas cinq à sept ans, mais le pronostic varie selon les patients: certains décèdent en quelques mois, d´autres, au contraire, ont une survie se prolongeant au-delà de dix ans [4]. L´objectif de cette étude est de décrire les particularités épidémiologiques cliniques, biologiques et pronostiques d´une série de patients ayant un myélome multiple colligés au Service des Laboratoires de l´Hôpital Habib Thameur de Tunis.

## Méthodes

**Conception de l'étude:** il s´agit d´une étude descriptive rétrospective portant sur une série de patients diagnostiqués au sein de l´Hôpital Habib Thameur de Tunis, sur une période de 10 ans, allant du 1^er^ janvier 2003 au 31 décembre 2023.

**Participants:** tous les patients ayant un dossier médical complet et dont l´analyse des critères cliniques, biologiques et radiologiques a permis de conclure au diagnostic et au suivi de MM ont été inclus. Les patients présentant une autre gammapathie monoclonale n´ont pas été inclus. Tous les patients ont bénéficié d´un bilan radiologique et biologique à visée diagnostique et pronostique.

**Variables:** les bilans biologiques à visée diagnostique comportaient l´hémogramme; la vitesse de sédimentation; le myélogramme; l´électrophorèse des protéines plasmatiques (EPP); l´immunofixation (IF) des protéines sériques et urinaires; la créatinine plasmatique et la calcémie (Ca). Une biopsie ostéomédullaire (BOM) avait été réalisée dans les cas douteux afin de confirmer le diagnostic de MM. Les investigations biologiques à visée pronostique comportaient le dosage de la protéine C-réactive (CRP) et la Bêta-2 microglobuline (β2m). Le diagnostic du myélome a été posé selon les critères de l´IMWG (2014) [5]. Au terme de ce bilan initial, les patients ont été classés selon la classification de Durie et Salmon [6]. Les données des dossiers ont été regroupées et intégrées dans une base de données informatique.

**Statistiques:** l´analyse des données a été réalisée en utilisant le logiciel Excel 2010.

## Résultats

### Caractéristiques générales de la population d´étude

Soixante (60) patients ont été inclus dans notre étude. Notre population était constituée de 26 hommes et de 34 femmes. Le sex-ratio était de 0,76. L´âge moyen de nos patients était de 67,61±8,7 ans, avec des extrêmes allant de 47 à 97 ans. Le délai médian entre le premier symptôme et le diagnostic était de 1 an (1 semaine-4 ans). Le motif principal de consultation était les douleurs osseuses dans 75% des cas (Tableau 1).

**Tableau 1:** circonstances de découverte au cours du myélome multiple

Motif d'admission	Nombre	Fréquence (%)
Douleurs osseuses	38	63
Altération de l'état générale	31	52
Syndrome anémique	4	7
Fracture pathologiques	4	7
Insuffisance rénale	1	2
Découverte d'un pic monoclonal	1	2
Déshydratation	1	2

### Aspects radiologiques

Les anomalies radiologiques étaient retrouvées dans 50 cas soit 83%. La lésion radiologique typique du MM est ostéolytique de siège différent surtout au niveau de la voûte crânienne, le bassin, les os longs et le rachis (Tableau 2).

**Tableau 2:** localisation des anomalies radiologiques

	Nombre	Fréquence (%)
Rachis	34	57
Crâne	25	42
Os longs	12	20
Bassin	9	15

### Aspects biologiques

#### Données hématologiques

L´anémie était observée dans 74% des cas, et elle était sévère (Hg<8,5g/dl) dans 25% des cas. L´anémie était normochrome normocytaire arégénérative dans 77% des cas, normochrome macrocytaire dans 14% des cas, et hypochrome microcytaire dans 9% des cas. L´hyperleucocytose et la leucopénie étaient respectivement retrouvées dans 13% et 10% des cas. La thrombopénie était retrouvée dans 17% des cas. Le myélogramme était réalisé chez tous nos patients. Il était concluant avec des plasmocytes supérieurs à 10% dans 70% des cas. La présence de plasmocytes dystrophiques a été notée dans 77% des cas. La BOM était réalisée dans 18 cas, car le myélogramme était non informatif. Elle a confirmé l´infiltration plasmocytaire dans tous les cas.

#### Données biochimiques

L´hyperprotidémie était retrouvée chez 35% des patients. Un pic monoclonal était retrouvé dans 87% des cas (Tableau 3). Une hypergammaglobulinemie bi-clonale était retrouvée chez un patient. Le tracé électrophorétique était normal chez trois patients (5%). L´IF des protéines était réalisé chez tous nos patients (Tableau 4). La chaine légère Kappa (K) était la plus dominante dans 67% des cas, avec prédominance de l´IgG Kappa (25%).

**Tableau 3:** caractéristiques biologiques

	Nombre	Fréquence(%)
Hémogramme		
Hémoglobine <12g/dL	44	74
Leucocytes <4000/mm^3^	6	10
Leucocytes >10000/mm^3^	8	13
Plaquettes <150/mm^3^	10	17
Infiltration plasmocytaire		
>60%	8	13
30-60%	10	17
10-30%	24	40
<10%	10	17
Vitesse de sédimentation>100mm	57	95
Paramètres biochimiques		
Protidémie > 80g/L	21	35
Albuminémie <35g/L	51	85
Pic monoclonal	52	87
Zone gamma	39	75
Zone bêta	12	23
Zone alpha 2	1	2
Calcémie>3mmol/L	4	7
Créatinémie>175 μmol/L	22	37

**Tableau 4:** répartition selon les résultats de l’immunofixation des protéines sériques

	Nombre	Fréquence (%)
Ig G	38	62
Ig A	13	22
Chaînes légères	7	12
Ig M	1	2
Ig G+Ig A	1	2
Ig: Immunoglobuline		

### Evolution et facteurs pronostiques

La classification de Durie et Salmon a répertorié nos patients comme suit: 74% stade III dont 37% au stade IIIa et IIIb respectivement, 13% stade II et 13% stade I. D´autres facteurs pronostiques ont été étudiés. Des taux élevés de CRP (>6mg/l) et de la β2m (>6mg/l) étaient retrouvés chez respectivement 55% et 33% de nos patients. L´insuffisance rénale était la complication la plus fréquente (37% des cas), suivi par les infections (32% des cas) et les manifestations neurologiques (13% des cas).

## Discussion

Le MM est une gammapathie monoclonale de diagnostic le plus souvent facile, fondé sur la présence d´une infiltration médullaire maligne, d´un pic monoclonal ou d´une atteinte organique. Il représente 10% des hémopathies malignes [7]. L´incidence du MM est en nette augmentation dans le monde où elle a été estimée à 7 nouveaux cas pour 100 000 habitants par an [8]. Dans ce travail, nous avons étudié les particularités cliniques, diagnostiques et pronostiques du MM chez 60 cas colligés au service de médecine interne du CHU Habib Thameur de Tunis.

### Aspects épidémiologiques

L´âge moyen de nos patients était de 67,61±8,7 ans avec des extrêmes allant de 47 à 97 ans et un pic de fréquence entre 60-69 ans, ce qui concorde avec les résultats des autres études [9,10].

Une prédominance féminine était notée dans notre série (sex-ratio H/F= 0.76). Ce résultat est similaire à ceux de plusieurs études [11,12].

D´autres études avaient retrouvé une prédominance masculine avec des sex-ratio respectifs de 1,62; 1,2 et 1,26 [9,13,14].

### Aspects cliniques

Dans notre série, le délai médian entre l´installation des premiers symptômes et le diagnostic du MM était de 12 mois. Selon Fall *et al*. [10], le délai entre le premier symptôme et le diagnostic du MM, évalué dans 136 cas, était en moyenne de 10 mois. Il était de 15 mois dans une étude de 59 cas réalisée dans le service de rhumatologie du CHU Sylvanus Olympio de Lomé [11]. Le long délai de consultation serait probablement lié au sous-développement ainsi qu´au caractère progressif de la maladie.

Les douleurs osseuses était la circonstance découverte la plus fréquente dans notre série (63% des cas) suivie de l´altération de l´état générale (52% des cas) ce qui concorde avec les résultats d´autres études [15,16].

### Aspects radiologiques

Les anomalies radiologiques étaient retrouvées dans 83% des cas. Elles étaient dominées par l´atteinte du rachis (57%) suivie par l´atteinte du crâne (42%) conformément aux données de la littérature [17].

### Aspects biologiques

#### Données hématologiques

Sur le plan biologique, la fréquence de l´anémie dans notre série était de 74%, elle était sévère (<8.5g /dl) dans 25% des cas. Nos résultats concordent avec les données des autres études [9,18]. Les causes de l´anémie sont multiples, parmi lesquels la prolifération plasmocytaire médullaire, une suppression de l´érythropoïèse induite par les cytokines, un phénomène d´hémodilution lié à l´hyperprotidémie et la diminution de sécrétion d´érythropoïétine en cas d´insuffisance rénale. L´hyperleucocytose et la leucopénie étaient respectivement retrouvées dans 13% et 10% des cas. L´hyperleucocytose n´est pas rare et elle est en rapport avec les complications infectieuses. Nos résultats se rapprochent de ceux rapportés dans d´autres études [18,19]. La thrombopénie aggrave le pronostic, elle est le reflet d´une importante masse tumorale. La thrombopénie était retrouvée dans 17% des cas. Les résultats retrouvés dans la littérature concernant la thrombopénie sont différents [18,19].

La VS à la première heure est le plus souvent élevée (>50 mm) ou très élevée (>100 mm). Ce phénomène est directement lié à la présence de la protéine monoclonale. Dans notre série tous nos patients ont une VS accélérée dont 95% était supérieur à 100 mm, ce qui concorde avec les résultats d´autres études [18,19]. Le myélogramme permet l´évaluation quantitative et qualitative de la plasmocytose médullaire, elle représente une étape décisive de la démarche diagnostique de MM. Cependant, il est parfois nécessaire de recourir à la BOM pour la confirmation du diagnostic à cause de la distribution hétérogène de la plasmocytose dans la moelle osseuse ou en cas de MM à moelle fibreuse. Dans notre série, le myélogramme a montré une moelle de bonne densité cellulaire, envahie à plus de 10% des plasmocytes chez 70% des patients. La présence de plasmocytes dystrophiques était notée dans 77% des cas.

Il s´agissait surtout de binucléarité, perte de l´excentricité du noyau, des plasmocytes vacuolés, des plasmocytes en mottes et des plasmocytes à cytoplasme flammé. Les plasmocytes étaient d´allure normale chez 23% des patients. La BOM était réalisée dans 18 cas, car le myélogramme était non informatif et a permis de confirmer le diagnostic dans tous les cas. Nos conclusions rejoignent les résultats de l´étude de Bouatay *et al*. [18], qui à l´examen du myélogramme, a montré la présence d´une infiltration plasmocytaire supérieure à 10% dans 76% des cas. La présence de signes de dystrophies cellulaires a été notée chez 80% des patients. La BOM a été réalisée dans 17 cas. Elle n´a pas pu confirmé l´infiltration plasmocytaire que dans 13 cas.

#### Données biochimiques

Dans notre série une hyperprotidémie était présente chez 66% des patients. Ce résultat rejoint ceux de la littérature [9,19]. Une hypoalbuminémie inférieure à 30 g/l témoigne d´une maladie avancée. Ce paramètre est corrélé à la masse tumorale [20]. Conformément à l´étude faite par Bouatay *et al*. [18] qui a trouvé une hypoalbuminémie chez 87% des patients, notre série a montré une albuminémie inférieure à 35g/l dans 85% des cas.

L´EPP avait montré un pic monoclonal dans 87% des cas: 75% des cas dans la zone gamma-globulines, 23% dans la zone bêta globulines et 2% dans la zone alpha2 globulines. Nos résultats sont similaires à ceux d´autres études [18,20]. L´EPP était normale chez 5% des patients. Ces résultats témoignent du manque de sensibilité de l´EPP pour la détection des gammapathies monoclonales notamment pour le MM à chaînes légères d´où l´intérêt de compléter par une immunoélectrophorèse et/ou une IF.

La particularité de notre étude était la mise en évidence d´un double pic à base étroite migrant dans la zone des gammaglobulines à l´EPP. Ce double pic constitue une situation rare de myélome multiple [21]. L´IF réalisée chez tous nos patients, avait montré différentes classes de gammapathie monoclonale: IgG dans 62 % des cas, IgA dans 22% des cas, IgM dans 2% des cas et chaînes légères chez 12%. Nous n´avons noté aucun cas de myélomes à IgD et aucun cas de myélome non excrétant. Nous avons en revanche noté un cas de myélome biclonal. Cette répartition de la fréquence des immunoglobulines rejoint globalement les données de la littérature avec quelques discordances dans certaines séries [10,18].

Le dosage de la calcémie fait partie des examens systématiques dans le bilan initial et la surveillance du MM. Dans notre étude, l´incidence de l´hypercalcémie était de 47% avec une hypercalcémie majeure dans 7% des cas. Les données de la littérature concernant l´hypercalcémie au cours du myélome sont diversifiés [18,19]. La prévalence de l´atteinte rénale au cours de l´évolution du myélome est de 30 à 50% selon les séries et la définition utilisée. Environ 30% des patients atteints de myélome multiple présentent une IR au moment du diagnostic. L´IR entraîne une morbi-mortalité importante chez ces patients. La néphropathie à cylindres myélomateux est la principale cause d´IR aiguë sévère liée au myélome multiple [22]. L´IR était présente chez 37% de nos patients. Ce résultat est conforme à ceux obtenus dans la littérature [18,23].

### Evolution et facteurs pronostiques

La classification de Salmon et Durie est la méthode de référence dans l´évaluation du pronostic pour la majorité des centres. C´est une classification qui est largement utilisée mais qui n´a de valeur qu´au moment du diagnostic, et qui ne prend pas en compte la cinétique de prolifération.

Nos patients étaient classés comme suit: 74% stade III, 13% stade II et enfin 13% stade III ce qui concorde avec les résultats d´autres études [18,24]. Cette prédominance du stade III au moment du diagnostic peut être expliquée par le retard de consultation et de diagnostic pour la majorité de nos patients. La β2m et la CRP sont des facteurs pronostiques indépendants liés à la masse tumorale. Il existe une corrélation entre leurs taux et la survie dans le MM [18].

Selon étude de Gaougaou *et al*. [9] la CRP et la β2m étaient augmentées chez, respectivement, 77% et 86% des patients. Dans notre étude la CRP était supérieure à 6 mg/l chez 55% et la β2m était supérieure à 6 mg/l chez 33% des cas. L´*ISS* (*International Staging System*) est un indice pronostique international qui tend à remplacer la classification de Salmon et Durie. Il est basé sur la combinaison de la β2m et l´albuminémie. Selon l´*ISS*, 33% de nos patients sont classés au stade III (médiane de survie 29 mois). Une révision de cette classification vient d´être adoptée par l´IMGW en 2015; elle ajoute aux anciens paramètres, le taux des LDH (Lactate déshydrogénase) et 3 types d´anomalies de haut risque détectées par la technique FISH des cellules plasmatiques. L´étude cytogénétique n´a pas été réalisée que chez 2 de nos patients.

Outre les anomalies cytogénétiques qui n´ont pas été prises en considération, notre étude a présenté d´autres limites. En raison des ressources médicales limitées, seuls les patients ayant un dossier complet ont été inclus menant à une taille faible d´échantillon (n=60). D´autre part, le caractère rétrospectif de l´étude n´a pas permis l´évaluation des autres facteurs pronostiques.

## Conclusion

Le MM est une gammapathie maligne caractérisée par le développement d'un clone de plasmocytes tumoraux envahissant la moelle hématopoïétique et sécrétant une immunoglobuline monoclonale. Le profil clinicobiologique des patients dans notre étude semble similaire à celui observé dans d'autres séries, où la majorité des patients sont classés au stade III selon la classification de Salmon et Durie. Cela souligne l´intérêt de développer des moyens de diagnostic précoces et d´étendre l´utilisation de la technique «FISH» pour l´analyse cytogénétique, afin de compléter l´évaluation pronostique des patients. Les paramètres pronostiques sont déterminants pour la sélection de la conduite thérapeutique adéquate et l´amélioration de la survie de chaque patient.

### Etat des connaissances sur le sujet

- Le myélome multiple (MM) est une dyscrasie plasmocytaire médullaire maligne liée à la sécrétion d´une immunoglobuline (Ig) monoclonale; c´est la plus fréquente des gammapathies malignes;
- Les manifestations polymorphes sont la base de critères diagnostiques qui ont été réactualisés en 2014.

### Contribution de notre étude à la connaissance

- La prédominance féminine rejoint les données de quelques études de la littérature;
- La particularité de notre étude était la mise en évidence d´un double pic à base étroite migrant dans la zone des gammaglobulines à l´EPP, ce double pic constitue une situation rare de myélome multiple;
- La majorité des patients dans notre étude sont classés au stade III selon la classification de Salmon et Durie; cela souligne l´intérêt de développer des moyens de diagnostic précoces et d´étendre l´utilisation de la technique « FISH » pour l´analyse cytogénétique, afin de compléter l´évaluation pronostique des patients.
